# Contract Negotiation Skills: A Workshop for Women in Medicine

**DOI:** 10.15766/mep_2374-8265.10910

**Published:** 2020-06-18

**Authors:** Amanda M. Simone, Melissa Simone, Lauren Block, Nancy LaVine

**Affiliations:** 1 General Internal Medicine Fellow, Department of Medicine, Donald and Barbara Zucker School of Medicine at Hofstra/Northwell; Physician, Internal Medicine, Allina Health; 2 Postdoctoral Research Fellow, Department of Psychiatry and Behavioral Sciences, University of Minnesota Medical School; 3 Associate Professor, Department of Medicine, Donald and Barbara Zucker School of Medicine at Hofstra/Northwell; 4 Assistant Professor, Department of Medicine, Donald and Barbara Zucker School of Medicine at Hofstra/Northwell

**Keywords:** Negotiation, Women in Medicine, Contract Negotiation, Role-Play, Professional Development, Faculty Development, Gender Issues in Medicine, Health Care Workforce, Mentoring/Coaching, Promotions and Tenure, Diversity, Inclusion, Health Equity

## Abstract

**Introduction:**

Contract negotiation is a high-stakes interaction, yet most physicians are never taught negotiation skills. Studies suggest that women, as compared with men, display a lower propensity to initiate negotiations and negotiate less competitively, highlighting a need for training to help level the playing field for female physicians.

**Methods:**

We devised a learner-centered workshop for female physicians that included a mini-didactic on negotiation principles, a question-and-answer time with a lawyer, an interactive role-play on contract negotiation style, and guided reflection. The workshop was intended for women in medicine from the level of medical student to full professor. The workshop was evaluated by pre- and postworkshop surveys with quantitative questions assessing perceived comfort with and knowledge of negotiation skills and strategies, as well as qualitative questions assessing lessons learned and areas for improvement.

**Results:**

After the workshop, participants (*n* = 34) reported significantly improved comfort with contract negotiation (*p* < .01) and with negotiation skills and strategies (*p* < .01). Through qualitative evaluation, we discovered that participants gained an appreciation for the self-advocacy in negotiation, as well as a better understanding of negotiation logistics. We also received positive feedback from participant comments, with most learners reporting that the topic was useful and worthwhile.

**Discussion:**

We believe that this workshop fills a gap in the literature regarding contract negotiation training for physicians while also helping to level the playing field with regard to female physicians and the gender pay gap.

## Educational Objectives

By the end of this workshop, participants should be able to:

1. Explain key negotiation microskills and terminology.
2. Identify strengths and areas for improvement in their current negotiation toolbox.
3. Conceptualize how to apply their preexisting negotiation skills to new negotiations.
4. Describe the role gender plays in negotiation.
5. Apply new skills in a role-play dealing with contract negotiation style.

## Introduction

The issues of gender bias and equity for women in medicine have come under scrutiny recently due to the gender pay gap,^1^ the disparity between women and men holding leadership positions within health care institutions,^1,2^ and the overall lower rate of women entering careers in academic medicine compared to their male counterparts.^3^ Progress being made in these areas is encouraging, with position papers and task forces focused on women in medicine being developed at many prominent health care organizations.^4,5^ We believe one of the reasons for this underlying pay gap is a lack of opportunity to develop skills in contract negotiation.

This disparity in negotiation skills between men and women is not limited to the medical field. As highlighted by Babcock and Laschever, women initiate negotiations less often than men in many fields, leading to women starting out behind their male counterparts in regard to salary and other job-related support.^6^ Not surprisingly, a recent meta-analysis revealed women also tend to achieve worse economic outcomes through negotiation.^7^ Furthermore, women more frequently feel that their situations are not negotiable and therefore do not attempt to negotiate in situations where their male counterparts often will, perpetuating this gap.^8^ One study posited that women who do negotiate have the potential to make at least one million dollars more over the course of their careers compared to those who do not negotiate.^6^ This statistic highlights a clear impetus for women to be more proactive with negotiations. We hope this workshop can help bridge this gap and help women feel more comfortable to ask when at the Table.

**Table.**
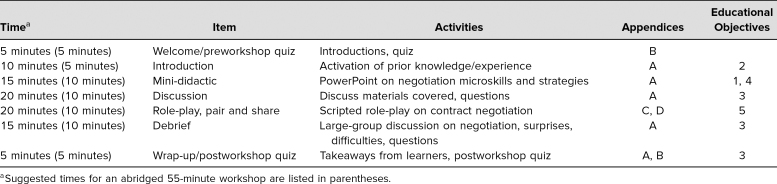
Suggested Time Line for Workshop

Through our literature review, we noticed scant evidence of published trainings or workshops on contract negotiation skills for physicians and found no such workshop in *MedEdPORTAL,* highlighting an area of need. A recent study in *Academic Medicine* describing negotiation experiences of faculty who have received early career awards (e.g., the National Institutes of Health's K award series) revealed that those individuals who felt more comfortable negotiating their contract terms highlighted mentoring and past experiences with workshops and trainings as having increased their comfort with these activities.^8^

Given this information, we recognized an opportunity to provide a workshop training on negotiation skills, with a goal of empowering female physicians to become better negotiators in future job and contract negotiations through discussion and guided practice. Based on the principles of principled negotiation, rather than hard or soft negotiation skills, we developed key microskills to be used to become an effective negotiator.^9^ The workshop combined a short didactic framing talk on these negotiation microskills, an open discussion with experienced female physician faculty and a contract lawyer, and an interactive role-play and pair-and-share.

We drew on principles of andragogy,^10^ social learning theory,^11^ and behavioral learning theory^12^ to provide a learner-centered program focused on understanding negotiation theory and improving negotiation skills through guided practice and reflection. The principles of andragogy—particularly that adult learners draw upon past experience and learn by doing—helped lay the foundation of our workshop.^10^ We recognized that while physicians in general may not have ample experience in contract negotiation specifically, they use negotiation skills throughout the day, from interacting with patients and staff regarding plans of care to interacting with loved ones at home. We drew upon these past negotiation experiences as a jumping point to frame our discussion and workshop on contract negotiation skills. We applied social learning theory, the idea that learning is a social behavior that can happen through observation of others, to help frame the development of the role-play portion of our workshop.^11^ Behavioral learning theory, which states that through reinforcement, learning leads to a change in behavior, also lent itself well to the use of a role-play and feedback in achieving the learning objectives of our workshop, especially that participants would leave the session with improved negotiation skills.^12^ After our session, we evaluated the workshop by comparing pre- and postworkshop surveys assessing learner comfort with contract negotiation and qualitative questions assessing lessons learned and areas for improvement.

### Workshop Piloting

This workshop was piloted twice prior to its official presentation at the Spring Women in Medicine Conference (SWIMC) at the Donald and Barbara Zucker School of Medicine at Hofstra/Northwell. It was first piloted with a group of four female internal medicine residents, at which time there was no lawyer present for discussion. Focus was placed on ensuring all participants got to complete a negotiation role-play. We received verbal feedback from these learners that there should be more time for discussion of negotiation strategies and past experiences. The workshop was next piloted and delivered as a noon-conference workshop for all internal medicine residents, PGY 1-PGY 3, at our institution, with an equal focus on both discussion and role-play. Feedback on this iteration was positive and relayed appreciation for training on the topic. We did receive the suggestion to include a lawyer or other contract expert who could give further insight into the legalities of contract negotiation, prompting us to add a lawyer for our final iteration of the workshop, given at the SWIMC.

## Methods

### Target Audience/Setting

This workshop was implemented at two sessions during the SWIMC. Our target audience included physicians of all training levels (from resident to full professor) who were attending the conference. The goal of the SWIMC was to educate female physician faculty about the promotion process, inspire these women to apply for career advancement, and connect female faculty from across specialties and experience levels.

We chose an interactive workshop model to enhance learner engagement and promote reflection and sharing. At our institution, the workshop was held in a conference room with tables set up for participants to work in groups of three or four, further facilitating engagement of the learners.

### Instructors

This workshop was facilitated by five presenters (two different pairs of physicians with the same lawyer at each session). The instructors included a physician with an understanding of negotiation skills and how gender bias plays a role in the health care workplace. It would be preferable for future instructors also to have experience with contract negotiations of their own, although this is not required. Additionally, the workshop calls for one instructor to be a lawyer or law student who has experience with contract law and can serve as a resource on the legal aspects of contract negotiation during a free-flowing question-and-answer session. This lawyer should be able to provide an overview of the rights of prospective physicians, address the legality of specific negotiation strategies, and offer a legal context for negotiation in general. If no lawyer or law student is available for future implementations, alternative facilitators could include a human resources representative or an institutional legal representative.

### Time Line

The session was intended to be 90 minutes in length. The Table outlines a suggested time line for the workshop, with suggestions for shortening the session to 55 minutes if necessary.

### Preparation

To prepare for this workshop, the facilitators reviewed the contract negotiation skills PowerPoint presentation (Appendix A) along with the notes, which contained suggested talking points for delivering the workshop. The survey, scripts, and checklists (Appendices B-D) were printed for each participant.

### Workshop Description and Resource Files

Pre- and postworkshop surveys (Appendix B) were given out to all participants during the introduction and again during the wrap-up. This tool allowed the facilitators to assess participant baseline negotiation knowledge and perceived comfort with negotiation prior to the workshop, as well as the effectiveness of the workshop on achieving the educational objectives at the end. The survey also contained qualitative questions, which allowed facilitators to collect feedback from participants on the workshop's content and usefulness.

After participants completed their preworkshop surveys, facilitators moved on to the introduction. Using the contract negotiation skills PowerPoint (Appendix A),^6-8,13-16^ the facilitators began a guided conversation as a way to get participants to begin to share their baseline knowledge of, and experience with, negotiation (slide 2). Then, the facilitators delivered a mini-didactic on negotiation microskills, styles, and strategies, as well as definitions of key negotiation terminology (slides 3–10). The PowerPoint notes section provided the facilitators with talking points and suggested language to use while discussing each slide.

After the mini-didactic, the facilitators led a large-group discussion on experiences with negotiation, including an open question-and-answer session with a lawyer regarding the legal aspects of contract negotiation (slide 11). The facilitators next had participants pair off to perform the role-play (slides 12–13). Each learner in a pair chose either the job applicant role or the employer role and performed script A (Appendix C). Then, participants switched roles and performed script B. Participants had approximately 5 minutes to role-play each script and received a 2-minute warning. Afterwards, both members of the pair used the negotiation checklist (Appendix D) to assess their own and their partner's performance.

After the role-play, participants were debriefed on their experiences. They were instructed to reflect on how it felt to negotiate during the role-play, what surprised them, and what questions they had (slides 14–15). The debriefing also provided a transition to the session wrap-up, at which time all participants shared one takeaway from the workshop (slide 16). After the wrap-up, the postworkshop survey (Appendix A) was given to all participants prior to the completion of the session.

### Workshop Evaluation

A mixed-methods study design was used to evaluate the session. Learners completed confidential pre- and postworkshop surveys, which included quantitative and qualitative items. These surveys were created by the workshop developers and refined through two rounds of piloting, as mentioned above. Learners answered Likert-type scale items assessing perceived comfort with contract negotiation and understanding of negotiation strategies. Learners also answered open-response questions asking what their favorite part of the workshop was, one thing they had learned, and one thing that remained unclear. There was also a space for any additional feedback or comments.

Surveys were analyzed using paired-sample *t* tests to compare pre- and postworkshop responses. Bivariate correlations were conducted to examine the extent to which survey responses were associated with comfort and understanding of negotiation strategies. For qualitative questions, we performed a thematic analysis using the bottom-up approach, consisting of open coding without a prespecified coding frame.^17^ Analyst triangulation was conducted by having two of the authors analyze the extracted quotations and develop and sort them into themes. Disputes in sorting were resolved by consensus, and a third author reviewed the final themes to look for consistency.^18^

## Results

A total of 34 learners participated in the workshop. We had a 100% response rate on our pre- and postworkshop surveys. All 34 participants identified as female. Most had not participated in negotiation prior to attending the workshop (73.5%). The majority of the sample reported that they were currently in residency (38.2%) or fellowship (20.6%). The remaining participants held the title of assistant professor (26.5%), associate professor (2.9%), or professor (8.8%). Participant years since completing graduate medical training ranged from 0 (still in residency) up to 27 years. Participants were from various medical specialties, including internal medicine, urology, neurosurgery, general surgery, psychiatry, and pediatrics.

The results from paired *t* tests revealed a significant improvement in how comfortable participants were with their ability to negotiate (presession average: 2.2, *SD* = 0.71, vs. postsession average: 3.5, *SD* = 0.71; *t*(33) = −7.32, *p* < .001). These scores were based on responses to a 6-point Likert-type scale (1 = *extremely uncomfortable,* 2 = *very uncomfortable,* 3 = *somewhat uncomfortable,* 4 = *somewhat comfortable,* 5 = *very comfortable,* 6 = *extremely comfortable*). The results also revealed a significant improvement in how well participants felt they understood negotiation strategies (presession average: 2.1, *SD* = 1.01, vs. postsession average: 3.9, *SD* = 0.78; *t*(33) = −8.17, *p* < .001). These scores were also based on a 6-point Likert-type scale (1 = *extremely unwell,* 2 = *very unwell,* 3 = *somewhat unwell,* 4 = *somewhat well,* 5 = *very well,* 6 = *extremely well*). Bivariate correlations revealed that years since completing training were not significantly correlated to comfort with negotiation on pre- or postsession survey responses (*r* = ±.01-.21, *p*s = .236-.993).

Themes identified from the question about the learner's favorite part of the workshop included the group discussion, the learning of new negotiation strategies, the role-play, and the legal aspects of negotiation.

From answers to the question about the most important thing learned in the workshop, identified themes included the power of self-advocacy in negotiation, the concept of negotiation a conversation, and an understanding of negotiation logistics. Representative comments for each of these themes included the following:

• The power of self-advocacy in negotiation:
  ○ “Never underestimate your value.”
  ○ “Advocate for myself.”
  ○ “Know your worth.”
• Negotiation is a conversation:
  ○ “Find a middle ground.”
  ○ “Negotiation is a give and take.”
• Understanding of negotiation logistics:
  ○ “What parts of a contract can be negotiated.”
  ○ “Negotiation microskills.”
  ○ “Get a lawyer to look at your contract.”

Themes from the question asking what learners felt was still unclear after the workshop included continued discomfort with the boundaries of contract negotiation, with comments such as “How far to push negotiation for what I want” and “Asking for salary transparency”; institution-specific questions, with “Our hospital's specific policies” as one response; and whether to negotiate relative value units (RVUs) versus salary, with one quotation being “Salary negotiation versus for RVU.”

Finally, from analysis of the feedback and comments question, two key themes emerged: overall praise for the session and recommendations for changes in workshop logistics, namely, the request for more time.

## Discussion

While outcomes of contract negotiations largely determine one's salary, benefits, and potentially even job satisfaction, there remains little training out there to help physicians improve their skills.^1,12,13^ The results of our workshop assessment reveal that a perceived lack of comfort with contract negotiation is not limited by experience, academic rank, or years in practice. Such results imply that training in this area is broadly useful and even necessary to help improve comfort and success in negotiation outcomes for providers of all levels.

In our results, we found through qualitative analysis that participants had an appreciation for all key components of the workshop, including the group discussion, mini-didactic, role-play, and legal aspects. Learners described an appreciation for the open discussion piece of the workshop, where they were able to ask questions freely, of both facilitators and other learners in the group. The open and collaborative nature of the workshop, participants reported, led to realizations that they were not alone in their struggle with feeling comfortable with negotiation. Learners also reported an appreciation for having a safe space in which to practice negotiation skills while also being able to receive feedback and debriefing. These findings highlight the benefits of having run two pilot workshops prior to our final implementation to allow for a well-rounded workshop that addressed the needs of our learners.

In our quantitative data results, we did see a significant improvement in learners’ perceived comfort with negotiation and understanding of negotiation skills and strategies, providing evidence that our workshop did, in fact, achieve its learning objectives. While qualitative feedback and comments from learners at the SWIMC were largely given in the form of praise for a great workshop in a necessary topic area, our quantitative results (although significantly improved) did not produce many instances of learners rating themselves as extremely comfortable with contract negotiation after the session. This is likely because negotiation is a skill that requires continued practice and experience in order to develop comfort.

Our workshop and evaluation do have a few limitations. The workshop was given at the SWIMC, a conference focused on the promotion and success of female faculty. As a result, all learners at the session were female physicians from a single institution. While the learners were of varying academic ranks and experience levels, the fact that we received evaluations only from female providers might limit the generalizability of expected results with and impact on larger audiences or other professions. Our future plans include presenting this workshop to a larger audience at a national conference, where we will be able to assess a more diverse learner population. We also plan to develop a long-term follow-up method to help assess whether participants retain or use the skills from this workshop in subsequent contract negotiations.

Additionally, through qualitative analysis, we realized that learners wanted more time to practice these skills and discuss. Given that the workshop was held within the 55-minute time constraints of a conference slot, we were unable to lengthen it there, but we do recommend the 90-minute version in order to provide ample time for discussion and practice in the form of role-play.

We believe that this workshop fills a gap in the literature regarding contract negotiation training for physicians. It has been well documented in multiple studies that there is a gender pay gap^1^ and that females consistently tend to negotiate for lower economic outcomes compared to their male counterparts.^12,13^ What is lacking, though, is an answer to this issue and, more importantly, a plan to help solve this problem. By using this workshop, which delivers training in negotiation strategies and dedicated practice time, we hope to start taking steps towards bridging that gap. Ultimately, this workshop, in our opinion, serves as a step in the right direction by starting a conversation, prompting practice in the form of role-plays, and making people more comfortable to continue to hone their negotiation skills.

## Appendices


Contract Negotiation Skills.pptxPre-Postworkshop Survey.docxRole-Play Scripts.docxRole-Play Checklist.docx
*All appendices are peer reviewed as integral parts of the Original Publication.*
