# Supplementary material for: Contract Negotiation Skills: A Workshop for Women in Medicine
Source: MedEdPORTAL. 2020 Jun 18;16:10910. doi: 10.15766/mep_2374-8265.10910 (PMC7331958; doi:10.15766/mep_2374-8265.10910)
Supplement: Supplementary file 1 — Contract Negotiation Skills.pptxPre-Postworkshop Survey.docxRole-Play Scripts.docxRole-Play Checklist.docx [file mep_2374-8265.10910-s001.zip › A. Contract Negotiation Skills.pptx]

## Slide 1
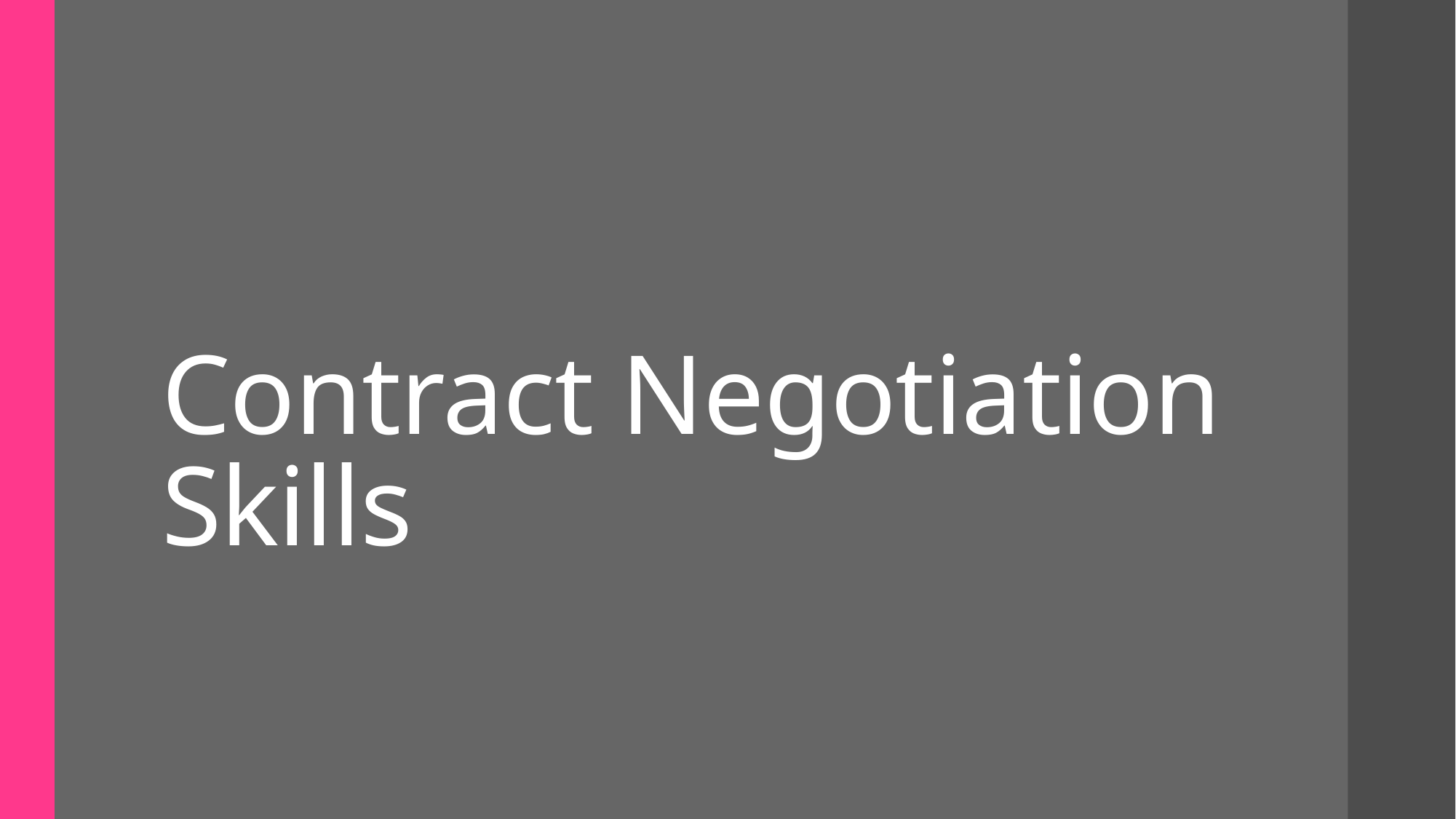

# Contract Negotiation Skills

## Slide 2
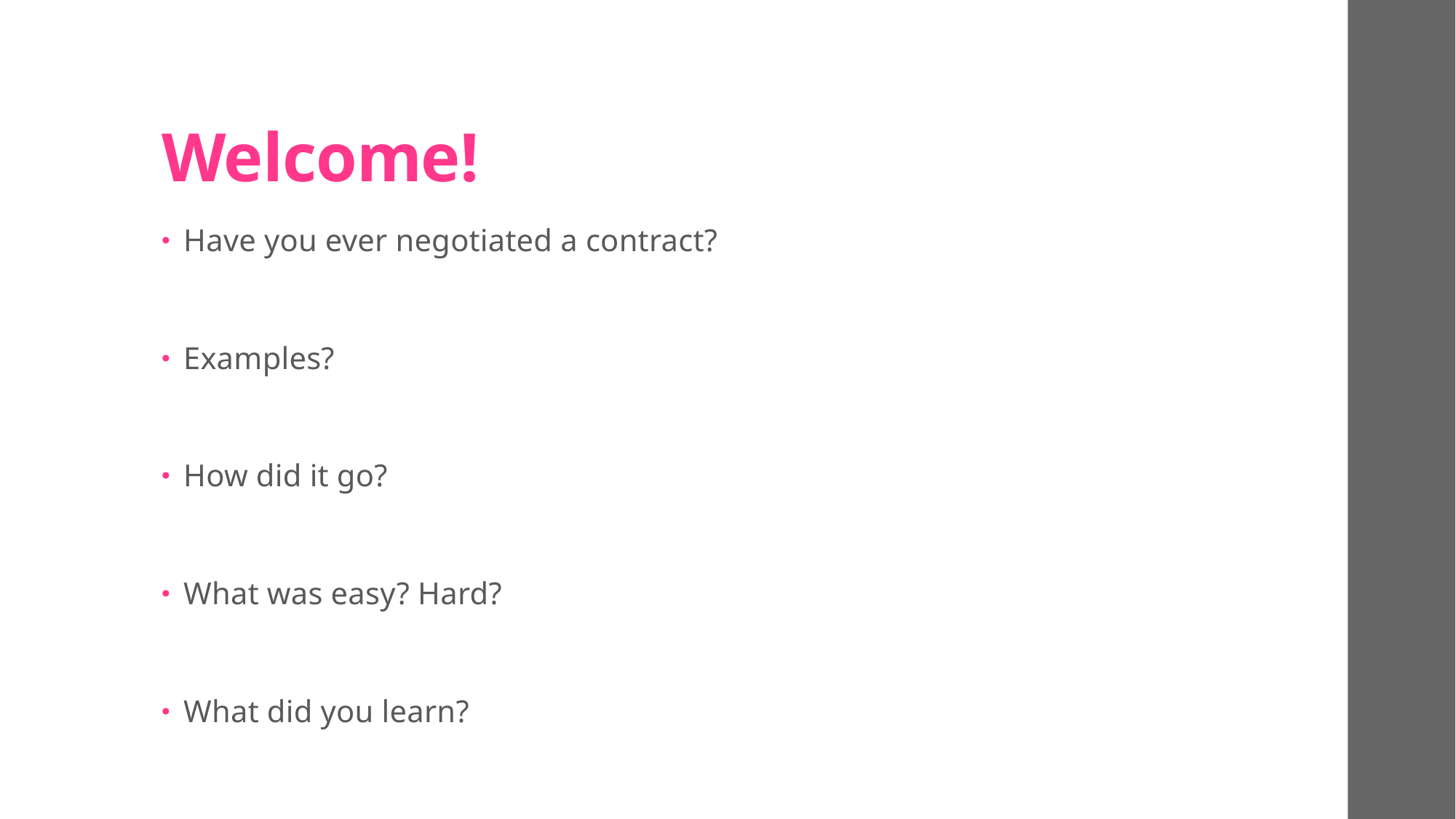

# Welcome!
Have you ever negotiated a contract?
Examples?
How did it go?
What was easy? Hard?
What did you learn?

## Slide 3
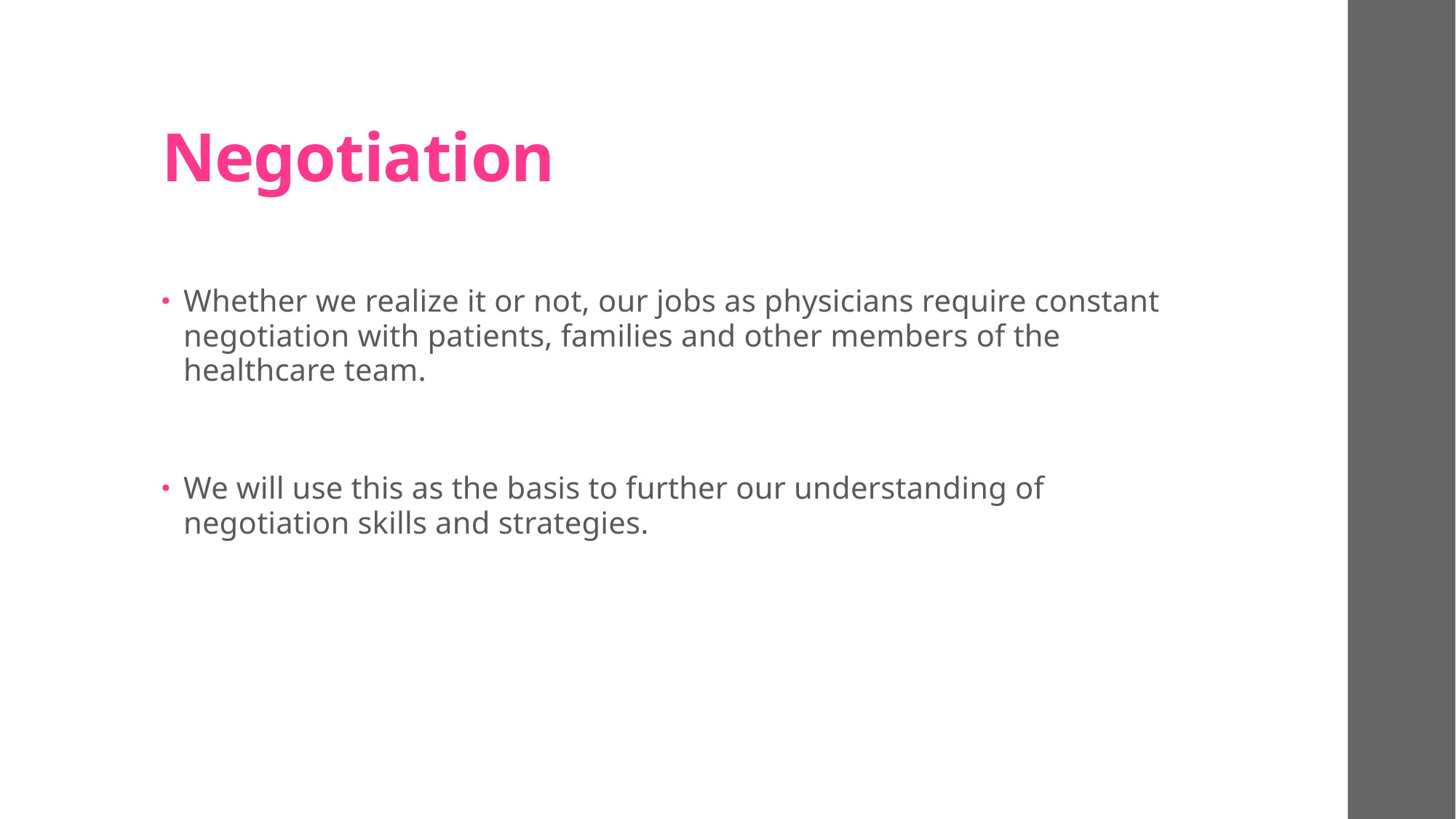

# Negotiation
Whether we realize it or not, our jobs as physicians require constant negotiation with patients, families and other members of the healthcare team.
We will use this as the basis to further our understanding of negotiation skills and strategies.

## Slide 4
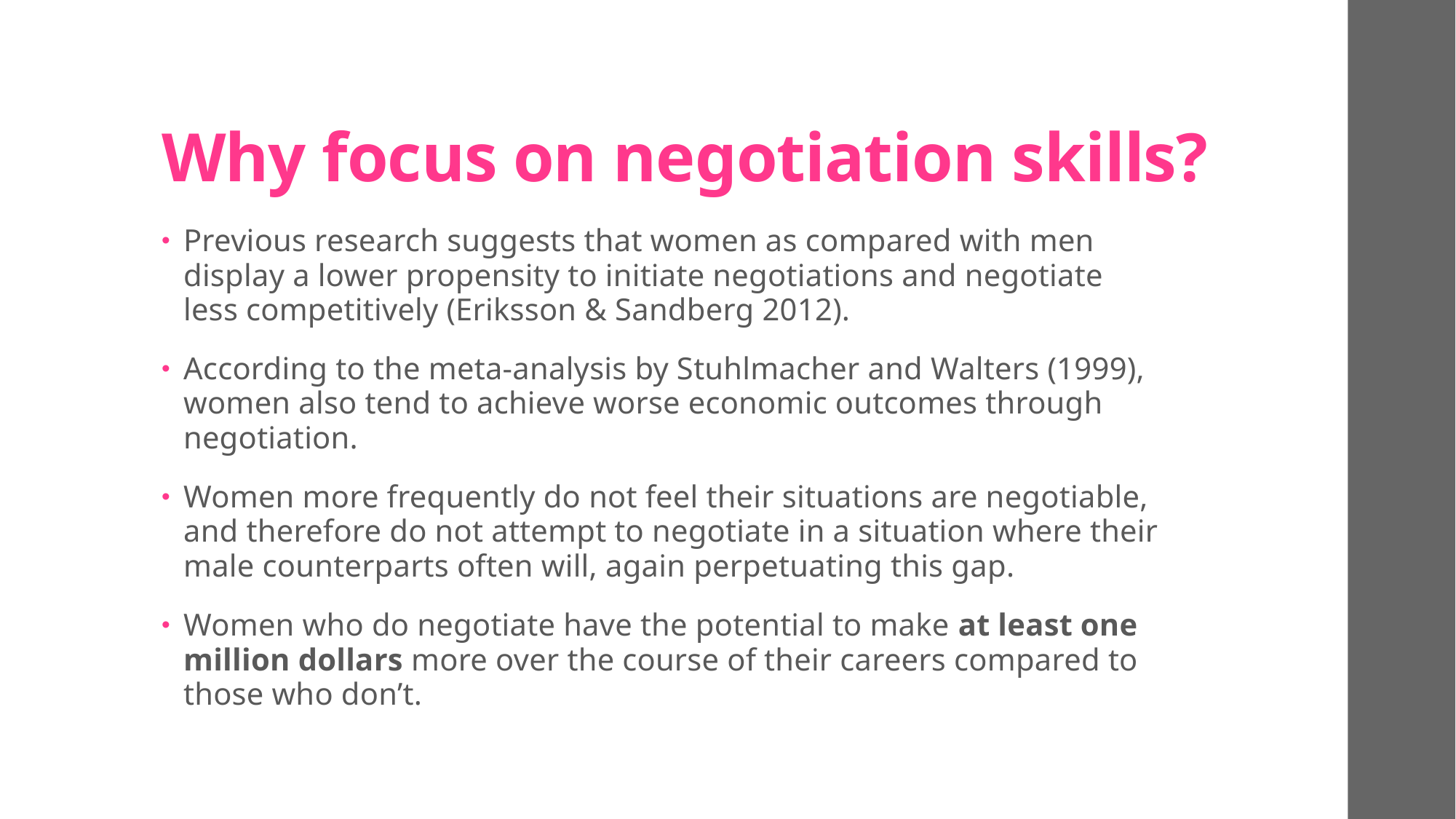

# Why focus on negotiation skills?
Previous research suggests that women as compared with men display a lower propensity to initiate negotiations and negotiate less competitively (Eriksson & Sandberg 2012).
According to the meta-analysis by Stuhlmacher and Walters (1999), women also tend to achieve worse economic outcomes through negotiation.
Women more frequently do not feel their situations are negotiable, and therefore do not attempt to negotiate in a situation where their male counterparts often will, again perpetuating this gap.
Women who do negotiate have the potential to make at least one million dollars more over the course of their careers compared to those who don’t.

## Slide 5
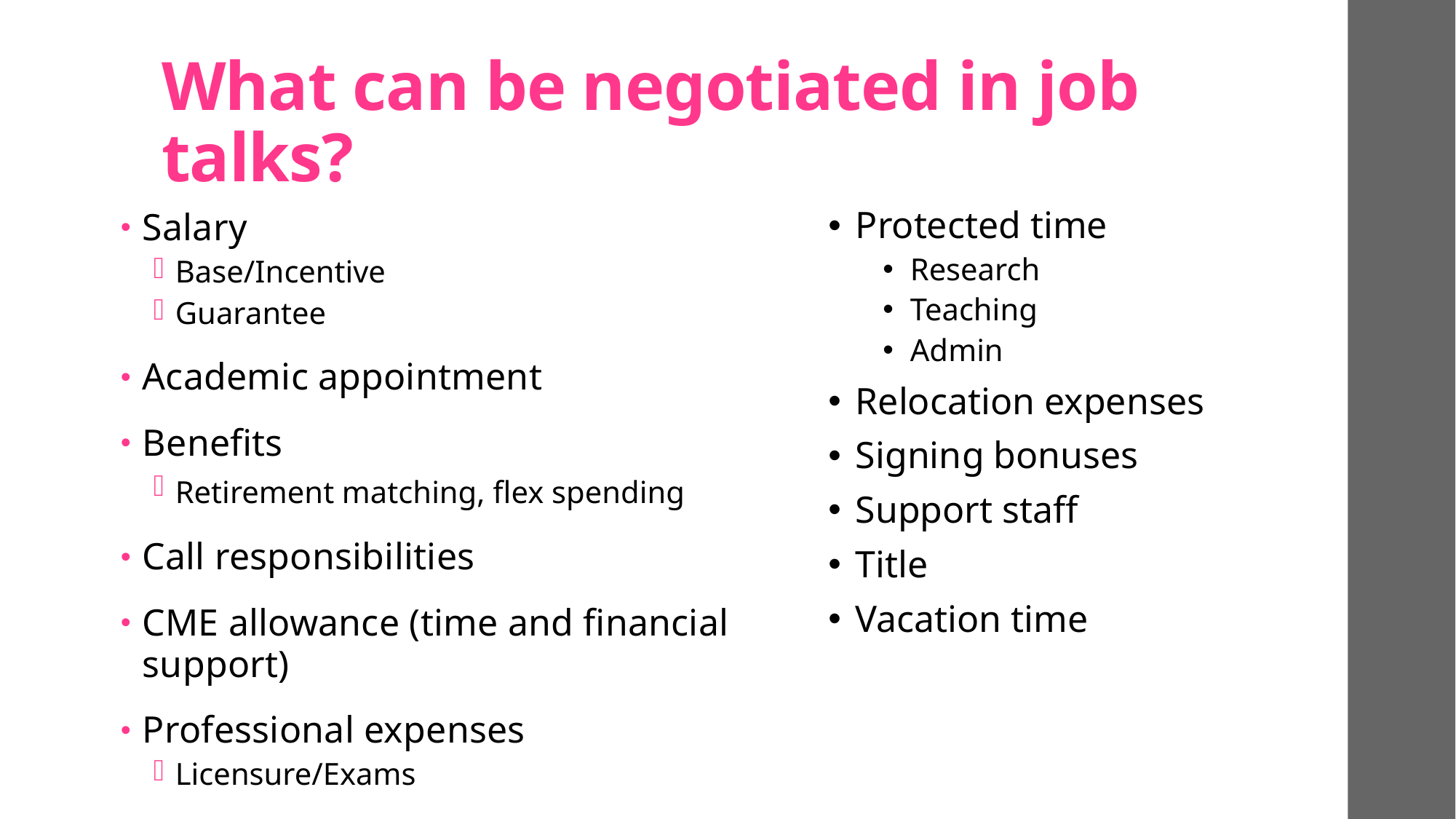

# What can be negotiated in job talks?
Salary
Base/Incentive
Guarantee
Academic appointment
Benefits
Retirement matching, flex spending
Call responsibilities
CME allowance (time and financial support)
Professional expenses
Licensure/Exams
Protected time
Research
Teaching
Admin
Relocation expenses
Signing bonuses
Support staff
Title
Vacation time

## Slide 6
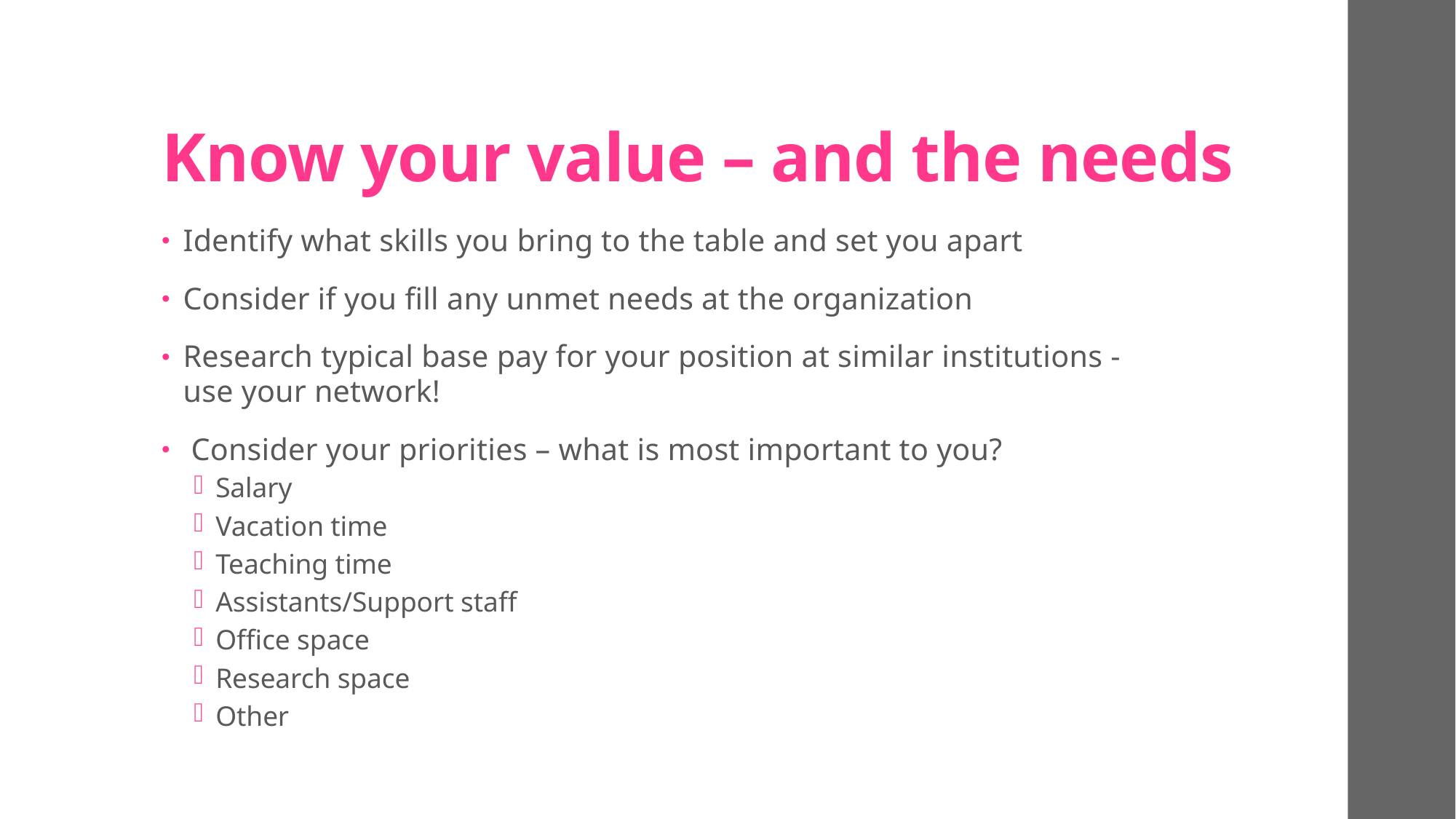

# Know your value – and the needs
Identify what skills you bring to the table and set you apart
Consider if you fill any unmet needs at the organization
Research typical base pay for your position at similar institutions - use your network!
 Consider your priorities – what is most important to you?
Salary
Vacation time
Teaching time
Assistants/Support staff
Office space
Research space
Other

## Slide 7
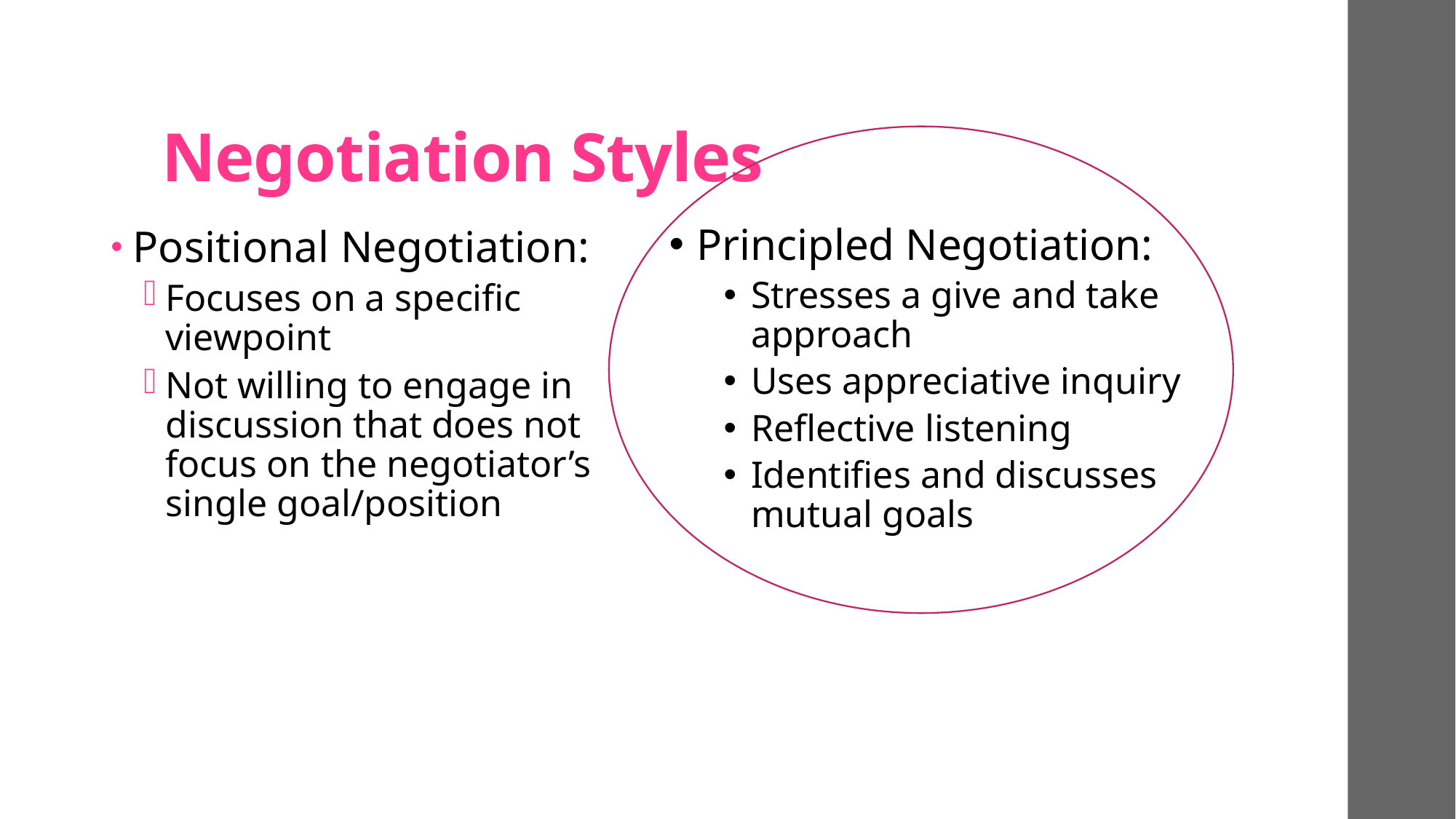

# Negotiation Styles
Positional Negotiation:
Focuses on a specific viewpoint
Not willing to engage in discussion that does not focus on the negotiator’s single goal/position
Principled Negotiation:
Stresses a give and take approach
Uses appreciative inquiry
Reflective listening
Identifies and discusses mutual goals

## Slide 8
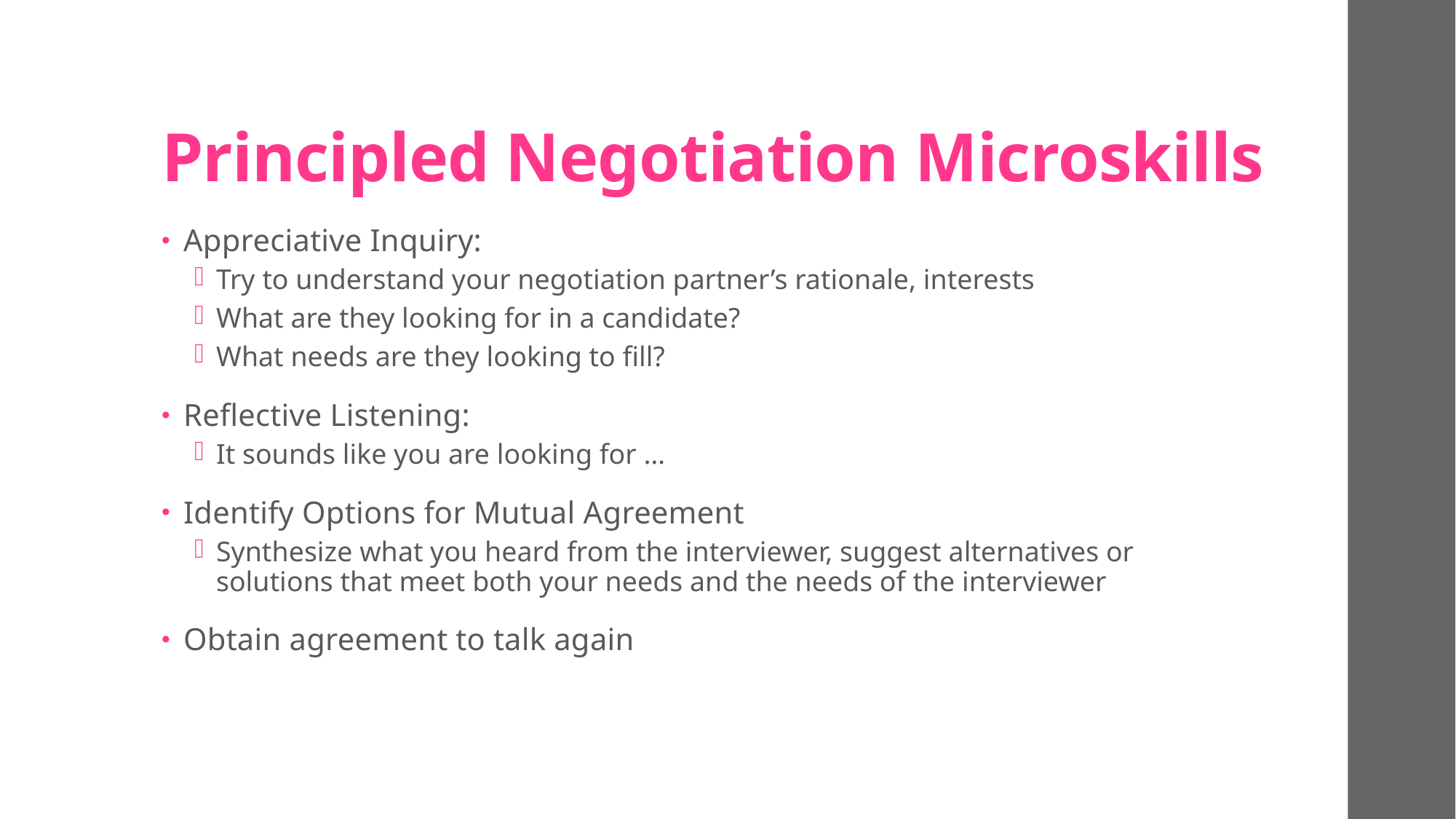

# Principled Negotiation Microskills
Appreciative Inquiry:
Try to understand your negotiation partner’s rationale, interests
What are they looking for in a candidate?
What needs are they looking to fill?
Reflective Listening:
It sounds like you are looking for …
Identify Options for Mutual Agreement
Synthesize what you heard from the interviewer, suggest alternatives or solutions that meet both your needs and the needs of the interviewer
Obtain agreement to talk again

## Slide 9
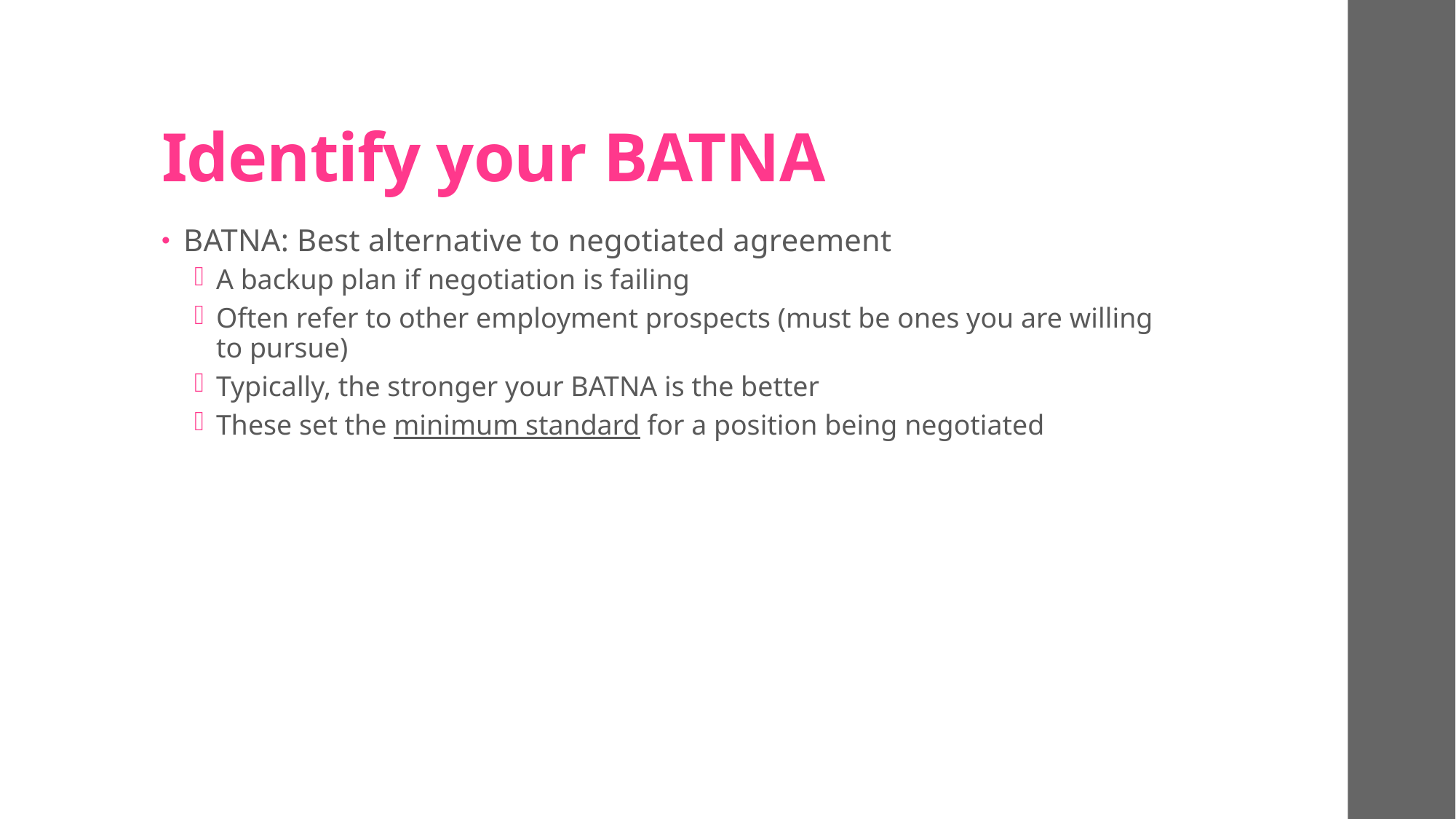

# Identify your BATNA
BATNA: Best alternative to negotiated agreement
A backup plan if negotiation is failing
Often refer to other employment prospects (must be ones you are willing to pursue)
Typically, the stronger your BATNA is the better
These set the minimum standard for a position being negotiated

## Slide 10
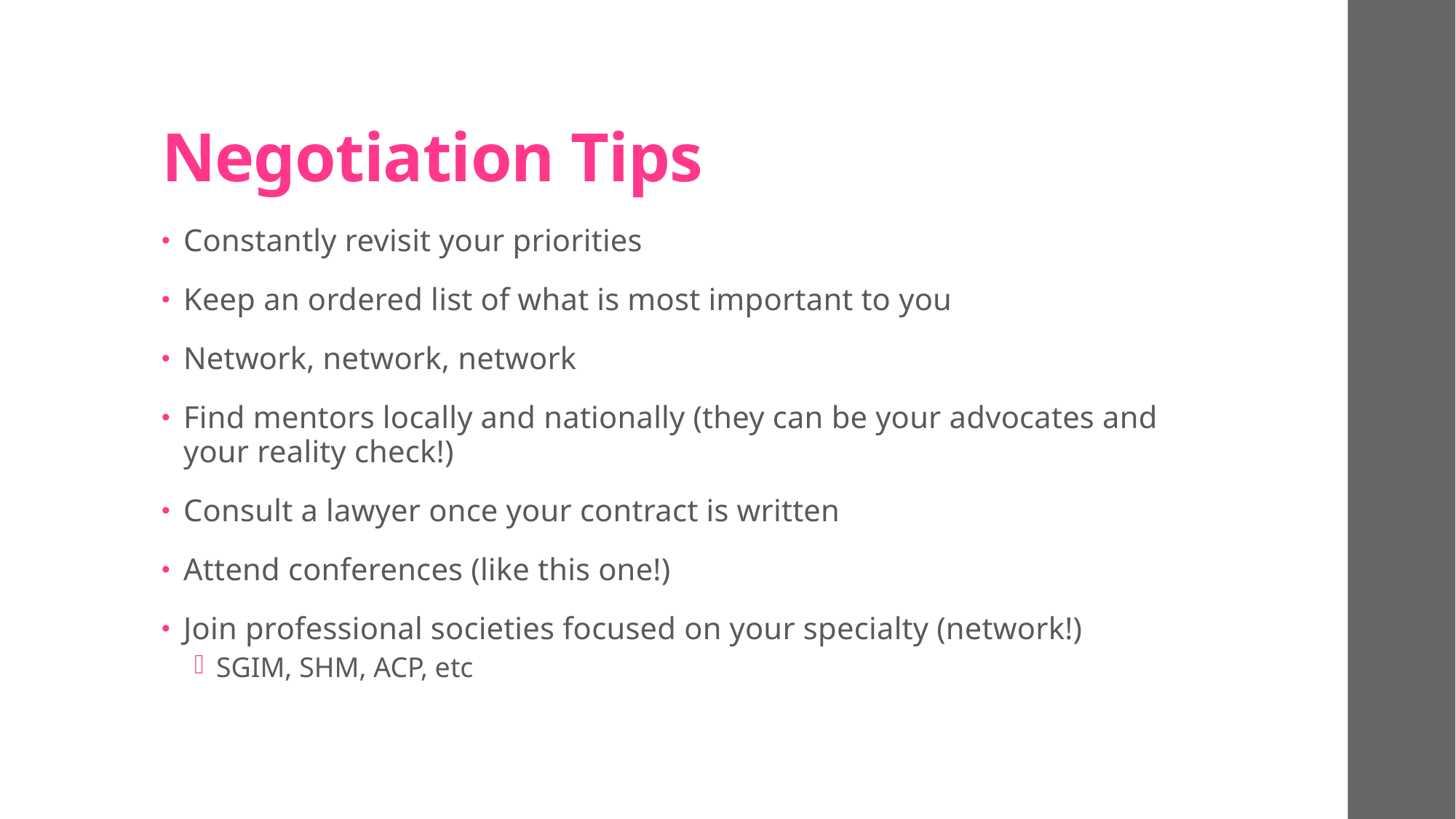

# Negotiation Tips
Constantly revisit your priorities
Keep an ordered list of what is most important to you
Network, network, network
Find mentors locally and nationally (they can be your advocates and your reality check!)
Consult a lawyer once your contract is written
Attend conferences (like this one!)
Join professional societies focused on your specialty (network!)
SGIM, SHM, ACP, etc

## Slide 11
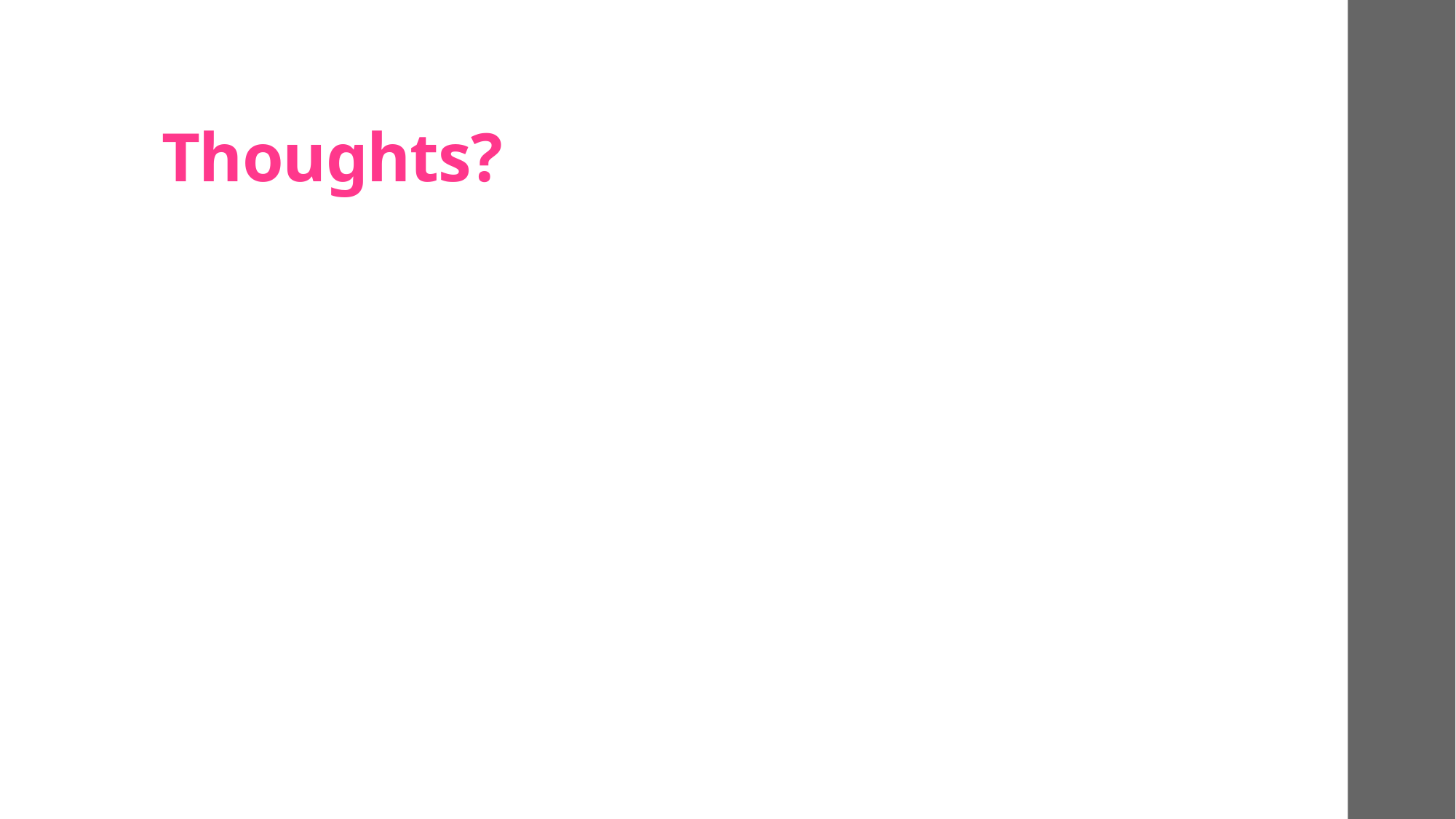

# Thoughts?

## Slide 12
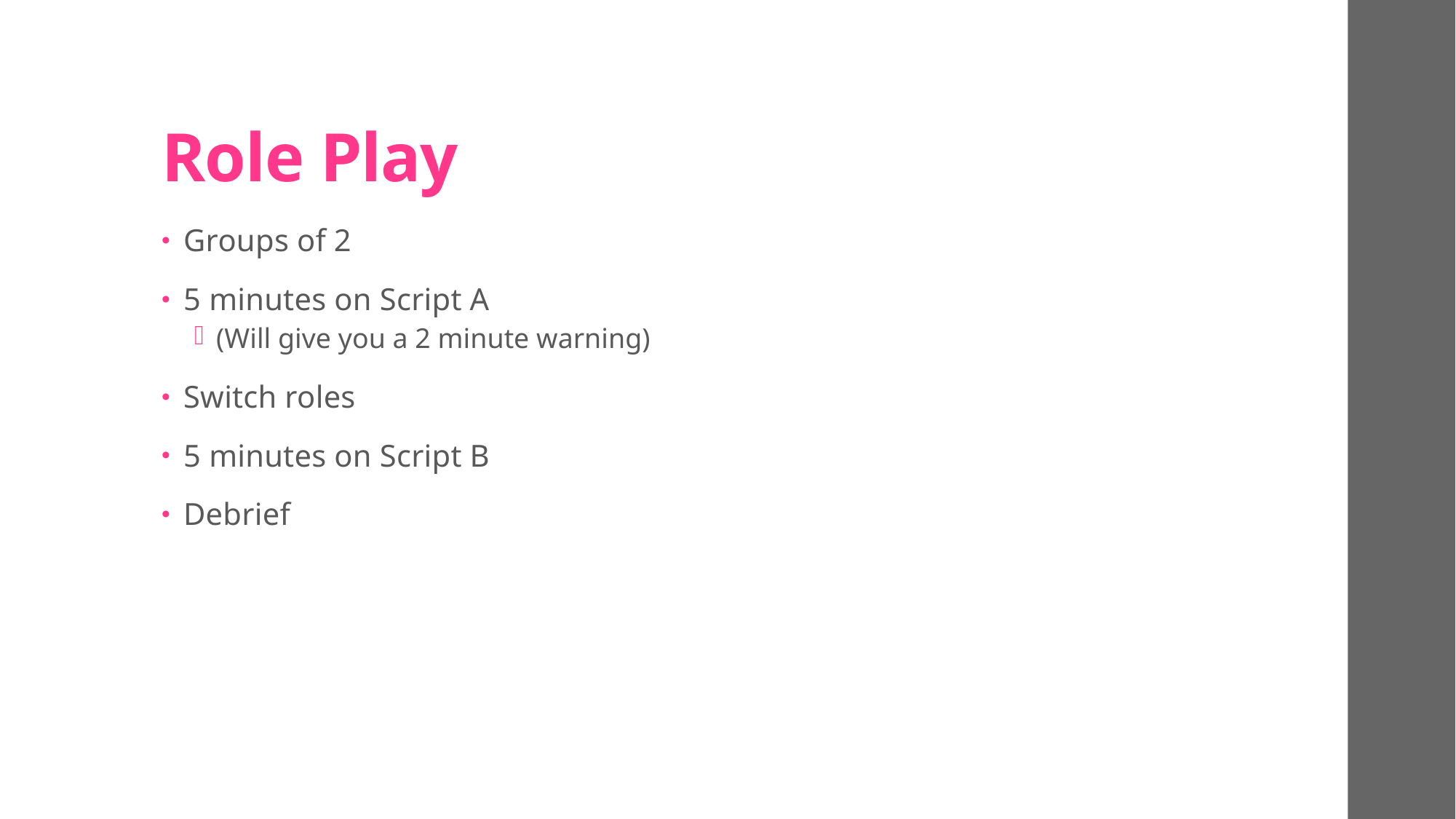

# Role Play
Groups of 2
5 minutes on Script A
(Will give you a 2 minute warning)
Switch roles
5 minutes on Script B
Debrief

## Slide 13
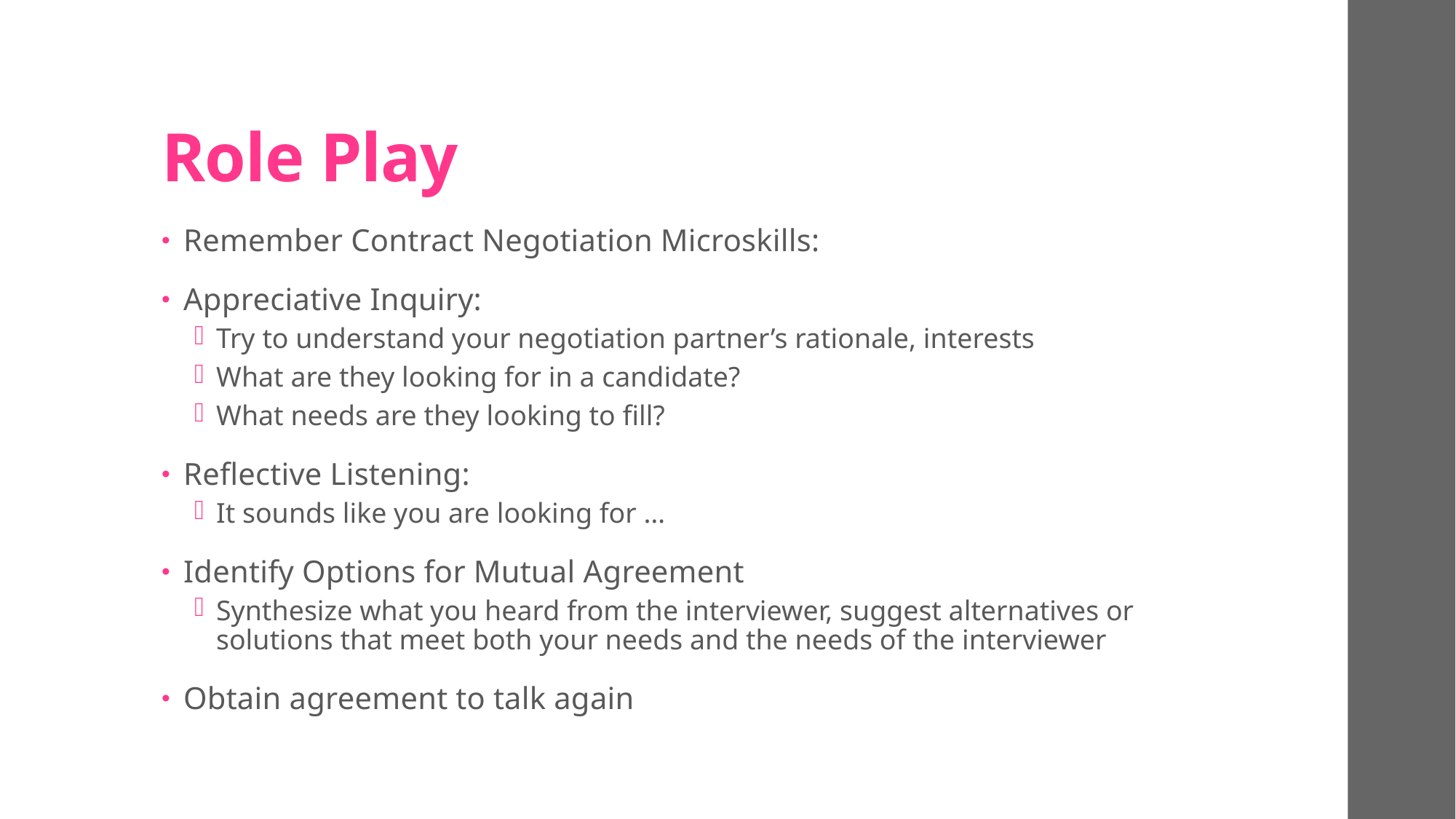

# Role Play
Remember Contract Negotiation Microskills:
Appreciative Inquiry:
Try to understand your negotiation partner’s rationale, interests
What are they looking for in a candidate?
What needs are they looking to fill?
Reflective Listening:
It sounds like you are looking for …
Identify Options for Mutual Agreement
Synthesize what you heard from the interviewer, suggest alternatives or solutions that meet both your needs and the needs of the interviewer
Obtain agreement to talk again

## Slide 14
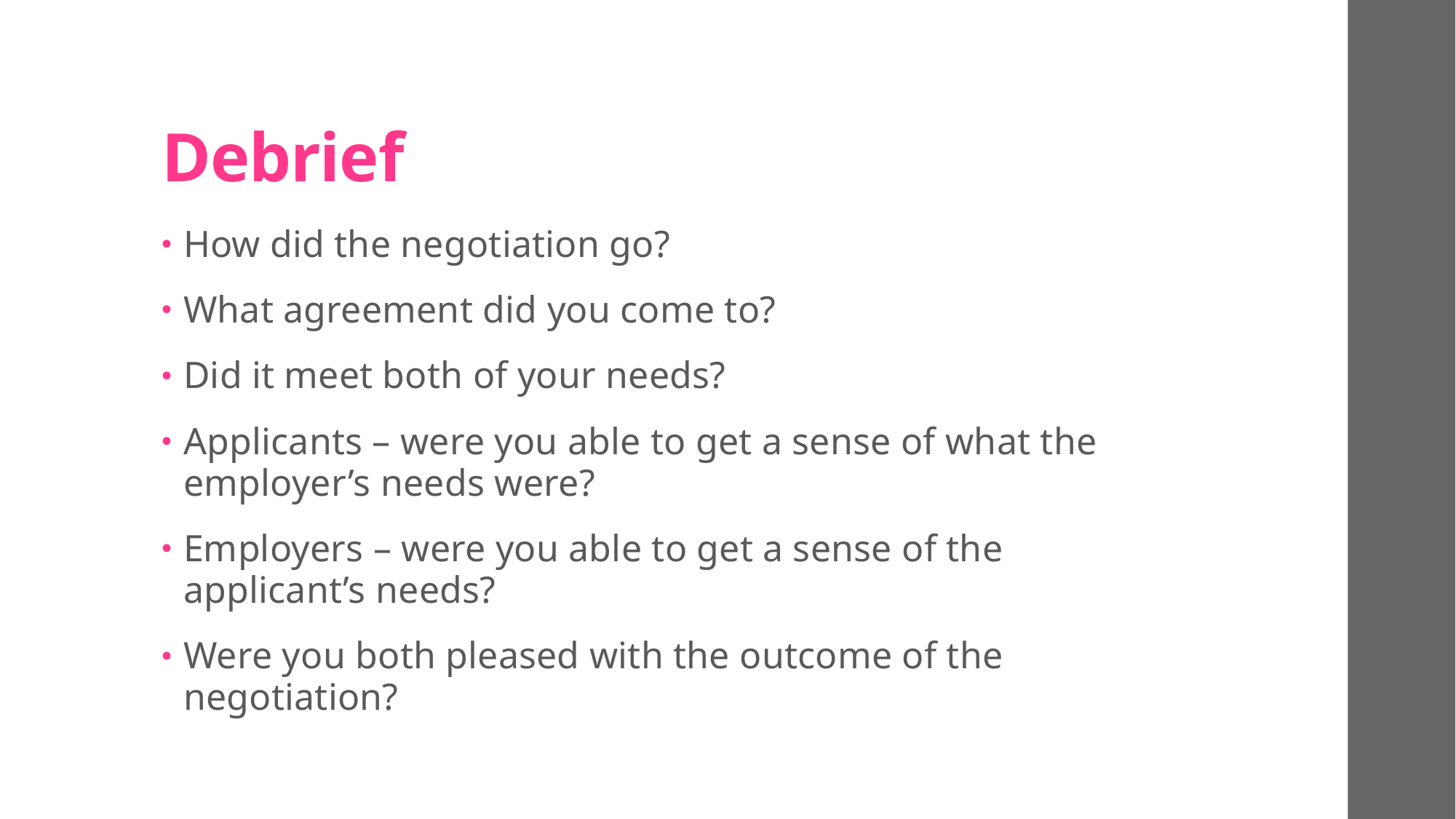

# Debrief
How did the negotiation go?
What agreement did you come to?
Did it meet both of your needs?
Applicants – were you able to get a sense of what the employer’s needs were?
Employers – were you able to get a sense of the applicant’s needs?
Were you both pleased with the outcome of the negotiation?

## Slide 15
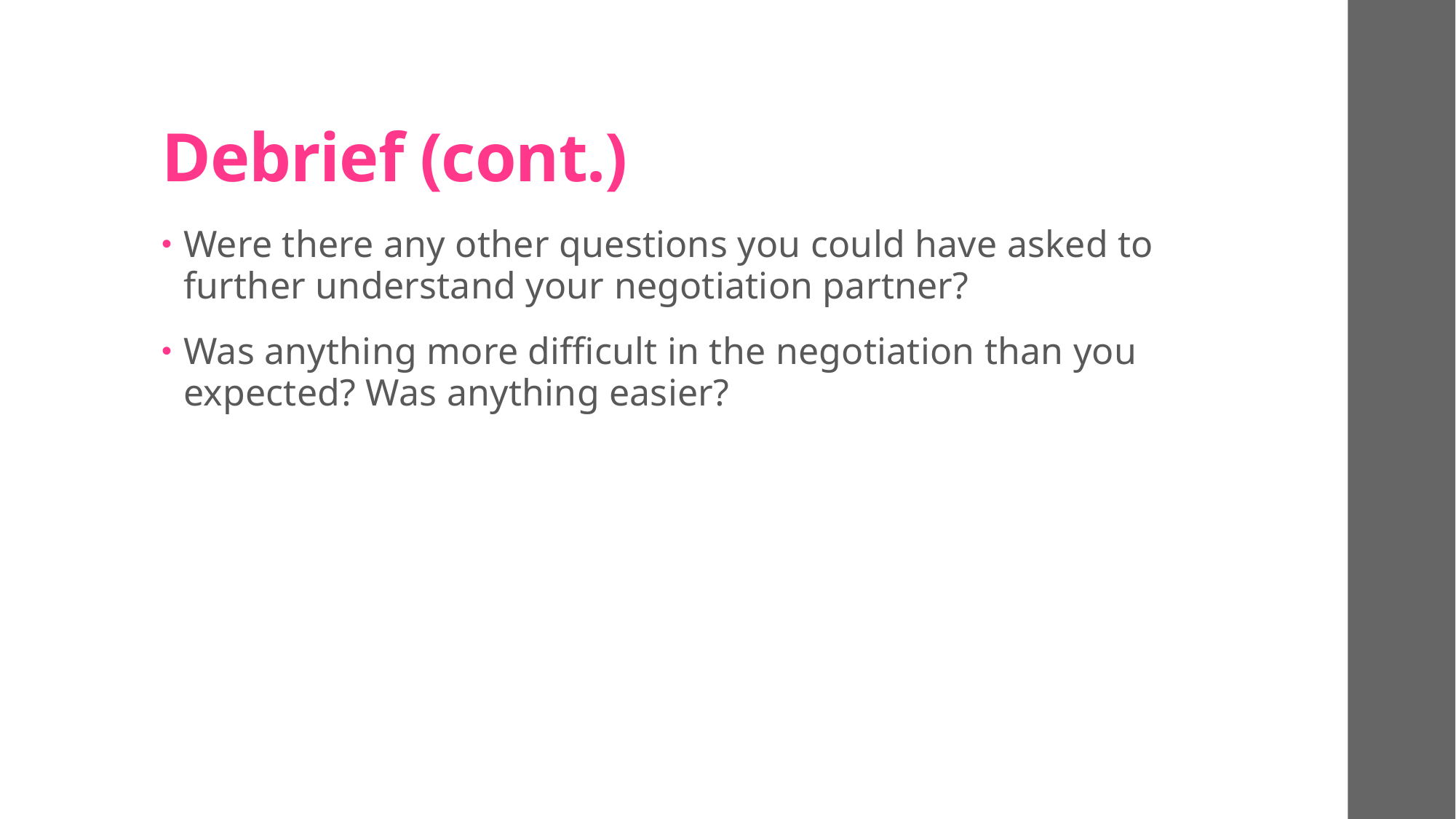

# Debrief (cont.)
Were there any other questions you could have asked to further understand your negotiation partner?
Was anything more difficult in the negotiation than you expected? Was anything easier?

## Slide 16
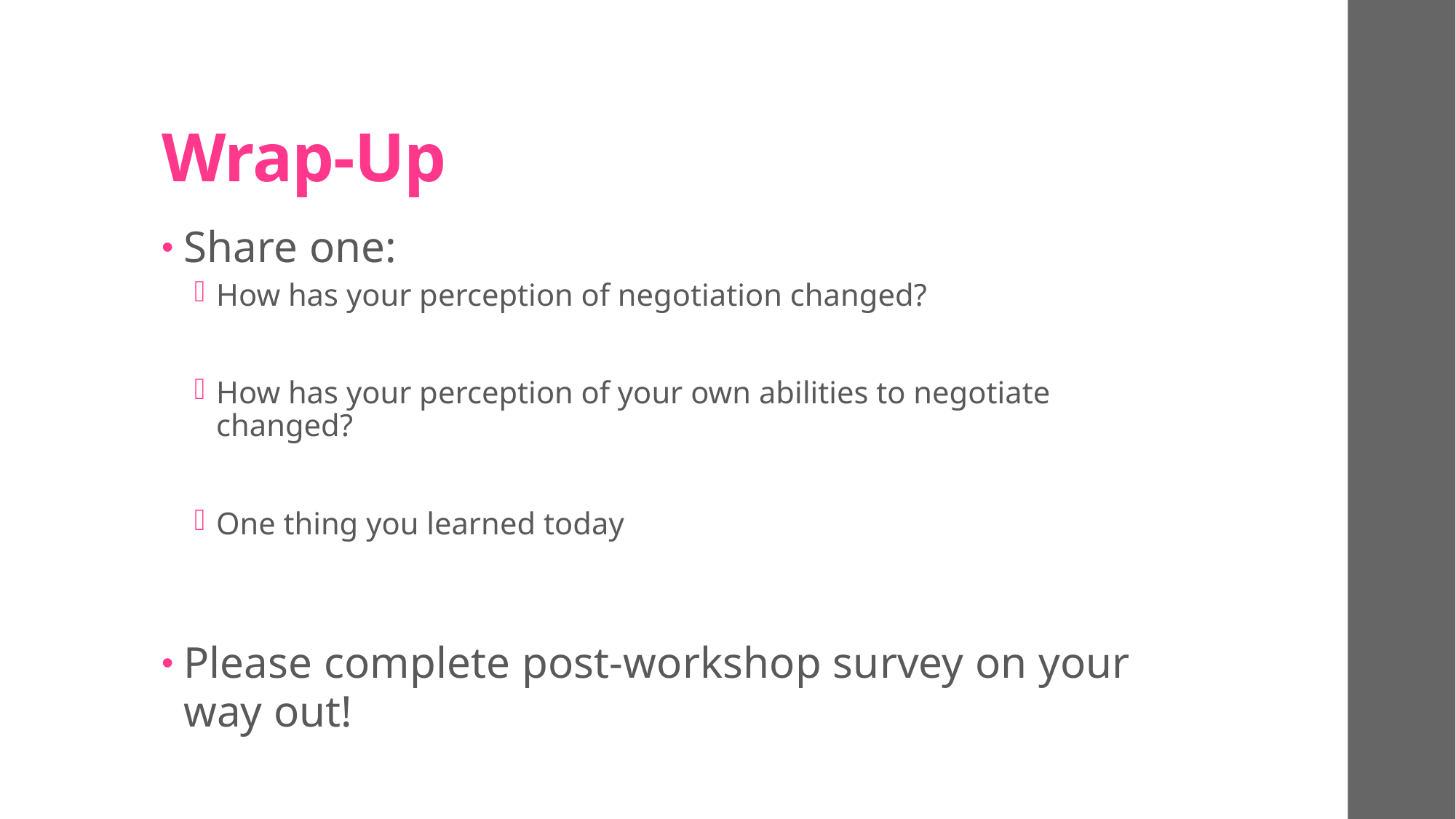

# Wrap-Up
Share one:
How has your perception of negotiation changed?
How has your perception of your own abilities to negotiate changed?
One thing you learned today
Please complete post-workshop survey on your way out!

## Slide 17
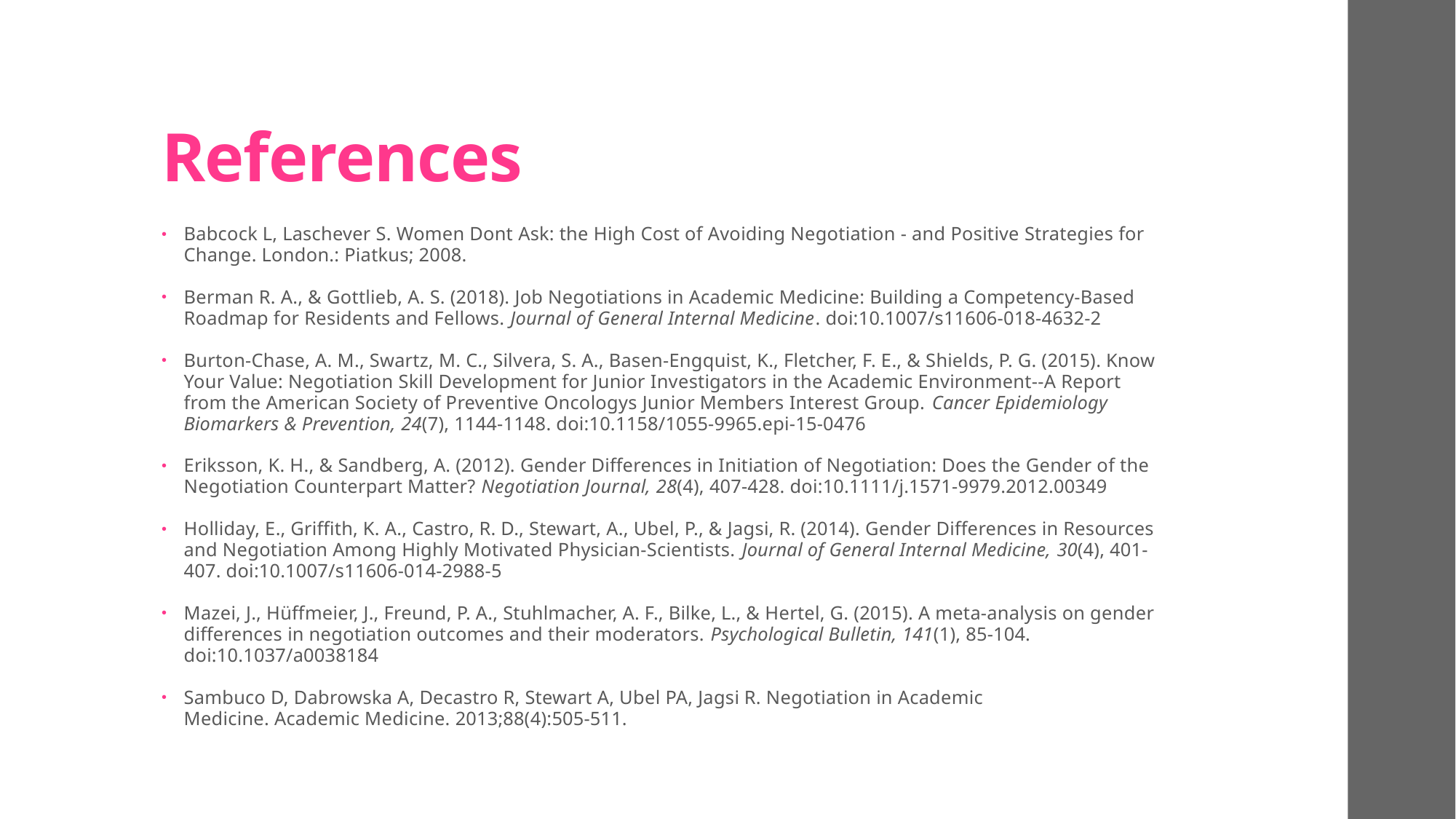

# References
Babcock L, Laschever S. Women Dont Ask: the High Cost of Avoiding Negotiation - and Positive Strategies for Change. London.: Piatkus; 2008.
Berman R. A., & Gottlieb, A. S. (2018). Job Negotiations in Academic Medicine: Building a Competency-Based Roadmap for Residents and Fellows. Journal of General Internal Medicine. doi:10.1007/s11606-018-4632-2
Burton-Chase, A. M., Swartz, M. C., Silvera, S. A., Basen-Engquist, K., Fletcher, F. E., & Shields, P. G. (2015). Know Your Value: Negotiation Skill Development for Junior Investigators in the Academic Environment--A Report from the American Society of Preventive Oncologys Junior Members Interest Group. Cancer Epidemiology Biomarkers & Prevention, 24(7), 1144-1148. doi:10.1158/1055-9965.epi-15-0476
Eriksson, K. H., & Sandberg, A. (2012). Gender Differences in Initiation of Negotiation: Does the Gender of the Negotiation Counterpart Matter? Negotiation Journal, 28(4), 407-428. doi:10.1111/j.1571-9979.2012.00349
Holliday, E., Griffith, K. A., Castro, R. D., Stewart, A., Ubel, P., & Jagsi, R. (2014). Gender Differences in Resources and Negotiation Among Highly Motivated Physician-Scientists. Journal of General Internal Medicine, 30(4), 401-407. doi:10.1007/s11606-014-2988-5
Mazei, J., Hüffmeier, J., Freund, P. A., Stuhlmacher, A. F., Bilke, L., & Hertel, G. (2015). A meta-analysis on gender differences in negotiation outcomes and their moderators. Psychological Bulletin, 141(1), 85-104. doi:10.1037/a0038184
Sambuco D, Dabrowska A, Decastro R, Stewart A, Ubel PA, Jagsi R. Negotiation in Academic Medicine. Academic Medicine. 2013;88(4):505-511.
