# Supplementary material for: Contract Negotiation Skills: A Workshop for Women in Medicine
Source: MedEdPORTAL. 2020 Jun 18;16:10910. doi: 10.15766/mep_2374-8265.10910 (PMC7331958; doi:10.15766/mep_2374-8265.10910)
Supplement: Supplementary file 1 — Contract Negotiation Skills.pptxPre-Postworkshop Survey.docxRole-Play Scripts.docxRole-Play Checklist.docx [file mep_2374-8265.10910-s001.zip › B. Pre-Postworkshop Survey.docx]

**Pre-workshop Survey:**

Last 4 digits of your cellphone number: __________

1. What is your current professional title and/or academic title?
2. How many years has it been since you completed residency training?
3. What is your gender?
   1. Female
   2. Male
   3. Other
   4. Prefer not to say
4. Have you participated in contract negotiations before?
   1. Yes (if yes, how many times? _____)
   2. No
5. If yes, how did the negotiation(s) go?
   1. Extremely well
   2. Very well
   3. Somewhat well
   4. Somewhat not well
   5. Very unwell
   6. Extremely unwell
6. How comfortable are you with your current ability to negotiate?
   1. Extremely comfortable
   2. Very comfortable
   3. Somewhat comfortable
   4. Somewhat uncomfortable
   5. Very uncomfortable
   6. Extremely uncomfortable
7. How well do you understand negotiation strategies?
   1. Extremely well
   2. Very well
   3. Somewhat well
   4. Somewhat not well
   5. Very unwell
   6. Extremely unwell

**Postworkshop Survey**

Last 4 digits of your cellphone number: ______________

1. How comfortable are you with your current ability to negotiate?
   1. Extremely comfortable
   2. Very comfortable
   3. Somewhat comfortable
   4. Somewhat uncomfortable
   5. Very uncomfortable
   6. Extremely uncomfortable
2. How well do you understand negotiation strategies?
   1. Extremely well
   2. Very well
   3. Somewhat well
   4. Somewhat not well
   5. Very unwell
   6. Extremely unwell
3. What change(s) do you plan to make in future contract negotiations after attending this workshop?
4. What was your favorite part of this workshop?
5. What is one thing you learned from this workshop?
6. What is one thing that is still unclear?

1. Any additional comments/feedback?
