# Supplementary material for: Contract Negotiation Skills: A Workshop for Women in Medicine
Source: MedEdPORTAL. 2020 Jun 18;16:10910. doi: 10.15766/mep_2374-8265.10910 (PMC7331958; doi:10.15766/mep_2374-8265.10910)
Supplement: Supplementary file 1 — Contract Negotiation Skills.pptxPre-Postworkshop Survey.docxRole-Play Scripts.docxRole-Play Checklist.docx [file mep_2374-8265.10910-s001.zip › C. Role-Play Scripts.docx]

**Appendix C**

Role-Play Scripts:

You are going to participate in a contract negotiation role-play. One of you will play the job applicant, and one will play the potential employer.

Below are two options (A & B) for contract negotiations. Chose one, read the background, and then role-play your negotiation as that person. In both cases, the applicant called the employer to discuss the contract. The applicant should start by introducing themselves.

**Script A**

**Job applicant:**

You are negotiating for a for a general internal medicine position at a highly-regarded academic healthcare institution in New York. You have been working in Connecticut as a successful clinician educator and internist for the last 5 years. This was your first job out of training. You are planning to leave your current job to relocate to NY where your family is.

The job you are applying for meets many of your needs. You will be able to continue your work as a clinician educator, receiving an appointment at the medical school, while still maintaining a primary care practice. The current offer is for 90% clinical time and 10% protected time for teaching, which they tell you is the starting format for all new hires. They tell you that this can shift “over time” to more teaching time. At your current job, you have 30% protected time for teaching, and would like to continue this (or even add more) in your new position. You recently revamped the physical diagnosis curriculum at your institution’s medical school, and you know that this is something of great value you can bring with you in your new job.

Your current job in Connecticut has offered to increase your base-pay if you stay, to exceed that which is being offered by this potential job, but you really do want to relocate to NY so long as you can get the teaching time you want.

Use your negotiation microskills to work towards a successful negotiation of teaching time with your potential employer.

**Potential Employer:**

You are the head of the GIM division a highly-regarded academic healthcare institution in New York. Your division has a commitment not only to excellent patient care, but also to the academic success and promotion of your physicians. You have an opening in the division for a primary care doctor that you are looking to fill. Typically, all new hires start with 90% clinical time with 10% of time protected for teaching or research. This is typically done so that clinicians can build up their practice and get comfortable with the flow without having other responsibilities pulling them out of the office. Despite this, you are open to negotiation of the schedule, as long as patient care and productivity is not sacrificed.

This applicant is a spectacular candidate, and you would love to have them join your department. They are calling you to discuss contract terms.

Use your negotiation microskills to work towards a successful negotiation with the job applicant.

**Script B**

**Job applicant:**

You are a graduating IM resident looking for jobs as a primary care physician with protected research time. You are about to negotiate a job offer from Spectacular Health University Hospital (SHUH) in Manhasset, NY. You are from Long Island and would love the opportunity to stay local with this job, as job location is one of your priorities. In your ideal job, you would work in the clinic 4 days a week and have 1 day protected for research time. The offer you received for this job at SHUH currently includes 5 full clinic days and no protected time for research. You are going to call the employer to negotiate a change in the clinic time in an attempt to gain 1 day of protected time for research.

You have been given a job offer at Upstate Health Center which offers your ideal schedule and identical salary, but is not as close to home as you would like.

Use your negotiation microskills to work towards a successful negotiation with your potential employer.

**Potential Employer:**

You are the head of the GIM division at the Spectacular Health University Hospital located in Manhasset, NY. Your division has a dedication to great patient care and also to high quality research. You have an opening in the division for a primary care doctor that you are looking to fill. Patient access has been suffering as two of your best practitioners recently left and office visits are now hard to come by for your patients. Your clinic serves many underserved patients, and your priority is to improve access to the clinic in any way possible. You ideally would like to hire a full-time clinical physician who can work 5 full days in the office seeing patients and thus aide in improving access to the office. You are open to negotiation of the schedule, as long as patient care and access is not sacrificed.

This applicant is a spectacular candidate, with evidence of a dedication to patient care and excellent recommendations, and you would love to have them join your department. They are calling you to discuss contract terms.

Use your negotiation microskills to work towards a successful negotiation with the job applicant.

**References**

1. Knowles MS. *The Adult Learner: a Neglected Species*. Houston, TX: Gulf; 1996.
2. Bandura A. *Social Learning Theory*. Englewood Cliffs, NJ: Prentice-Hall; 1977.
3. Lave J, Wenger E. *Situated Learning: Legitimate Peripheral Participation*. Cambridge, United Kingdom: Cambridge University Press; 2016.
4. Berman RA, Gottlieb AS. Job Negotiations in Academic Medicine: Building a Competency-Based Roadmap for Residents and Fellows. *Journal of General Internal Medicine*. January 2018. doi:10.1007/s11606-018-4632-2
