# Supplementary material for: Contract Negotiation Skills: A Workshop for Women in Medicine
Source: MedEdPORTAL. 2020 Jun 18;16:10910. doi: 10.15766/mep_2374-8265.10910 (PMC7331958; doi:10.15766/mep_2374-8265.10910)
Supplement: Supplementary file 1 — Contract Negotiation Skills.pptxPre-Postworkshop Survey.docxRole-Play Scripts.docxRole-Play Checklist.docx [file mep_2374-8265.10910-s001.zip › D. Role-Play Checklist.docx]

**Appendix D**

Negotiation Checklist:

Assess your negotiation role-play using the following checklist:

Did you use the following microskills during the negotiation?

| Skill | Yes | No | Comments |
| --- | --- | --- | --- |
| Appreciative Inquiry |  |  |  |
| Reflective Listening |  |  |  |
| Identification of items of mutual agreement |  |  |  |
| Agreement to talk again |  |  |  |

**References**

1. Knowles MS. *The Adult Learner: a Neglected Species*. Houston, TX: Gulf; 1996.
2. Bandura A. *Social Learning Theory*. Englewood Cliffs, NJ: Prentice-Hall; 1977.
3. Lave J, Wenger E. *Situated Learning: Legitimate Peripheral Participation*. Cambridge, United Kingdom: Cambridge University Press; 2016.
4. Berman RA, Gottlieb AS. Job Negotiations in Academic Medicine: Building a Competency-Based Roadmap for Residents and Fellows. *Journal of General Internal Medicine*. January 2018. doi:10.1007/s11606-018-4632-2
